# Network coordination following discharge from psychiatric inpatient treatment: a study protocol

**DOI:** 10.1186/1471-244X-13-220

**Published:** 2013-09-04

**Authors:** Agnes von Wyl, Gisela Heim, Nicolas Rüsch, Wulf Rössler, Andreas Andreae

**Affiliations:** 1 Department of Applied Psychology, Zurich University of Applied Sciences (ZHAW), Minervastrasse 30, P.O. Box Zurich CH-8032, Switzerland; 2 Integrated Psychiatric Clinic of Winterthur and Zurich Unterland (ipw) Wieshofstrasse 102, P.O. Box 144 Winterthur CH-8408, Switzerland; 3 Psychiatric University Hospital Zürich, Militärstrasse 8, P.O. Box 1930 Zürich CH-8021, Switzerland; 4 Department of Psychiatry II, University of Ulm, Ulm, Germany

**Keywords:** Post-discharge coordination, Randomized controlled trial (RCT), Social casework, Mental health care, Outpatient care, Discharge planning

## Abstract

**Background:**

Inadequate discharge planning following inpatient stays is a major issue in the provision of a high standard of care for patients who receive psychiatric treatment. Studies have shown that half of patients who had no pre-discharge contact with outpatient services do not keep their first outpatient appointment. Additionally, discharged patients who are not well linked to their outpatient care networks are at twice the risk of re-hospitalization. The aim of this study is to investigate if the Post-Discharge Network Coordination Program at ipw has a demonstrably significant impact on the frequency and duration of patient re-hospitalization. Subjects are randomly assigned to either the treatment group or to the control group. The treatment group participates in the Post-Discharge Network Coordination Program. The control group receives treatment as usual with no additional social support. Further outcome variables include: social support, change in psychiatric symptoms, quality of life, and independence in daily functioning.

**Methods/design:**

The study is conducted as a randomized controlled trial. Subjects are randomly assigned to either the control group or to the treatment group. Computer generated block randomization is used to assure both groups have the same number of subjects. Stratified block randomization is used for the psychiatric diagnosis of ICD-10, F1. Approximately 160 patients are recruited in two care units at Psychiatrie-Zentrum Hard Embrach and two care units at Klinik Schlosstal Winterthur.

**Discussion:**

The proposed post-discharge network coordination program intervenes during the critical post-discharge period. It focuses primarily on promoting the integration of the patients into their social networks, and additionally to coordinating outpatient care and addressing concerns of daily life.

**Trial registration:**

ISRCTN: ISRCTN58280620

## Background

In most Western countries, psychiatric care is undergoing deinstitutionalization. The main locus of care provision has shifted from inpatient to outpatient care settings. The number of psychiatric inpatient beds has been reduced, and the duration of psychiatric hospitalizations has decreased [1]. Due to increasingly shorter inpatient stays, structured discharge planning has become an urgent need. The first six weeks after discharge from inpatient psychiatric care represent a very stressful period for patients. There is evidence that suicide risk is greatly increased during this time, especially following a short psychiatric hospital treatment [2]. A study in the French-speaking part of Switzerland identified poorly planned discharges as one of the greatest problems in the treatment of persons with mental disorders [3]. Studies have shown that 50% of patients who had no communication with an outpatient clinician prior to discharge from inpatient care did not keep their first outpatient appointments [4,5]. Patients who are not linked to outpatient services have a doubled risk of re-hospitalization [6]. From a clinical perspective, several organizational measures are required to improve the linkage of patients with outpatient services. In 1997 Meisler et al. [7] named four important measures for ensuring continuity of care: (1) Inpatient clinic staff is responsible for the interface between inpatient and outpatient care; (2) The period of time between discharge and the beginning of outpatient care should be short; (3) A case manager must have the option to visit patients at home; and (4) Patients need more intensive care in the period immediately after discharge.

Indeed, subsequent clinical studies have demonstrated the effectiveness of systematic discharge planning for persons with mental disorders. In a meta-analysis of over 11 clinical studies, Steffen, Kösters, Becker, and Puschner [8] found that discharge planning interventions were effective in improving continuity of care and reducing symptom severity. The authors noted, however, that in many of the studies that they examined, the discharge strategies did not sufficiently comply with the quality standards of good clinical practice (i.e. at least one meeting of the patient and all individuals involved in the network prior to discharge from inpatient care). A multicenter randomized controlled trial (the NODPAM study) [9] carried out at psychiatric hospitals in Germany is currently investigating the effectiveness of a manualized needs-oriented discharge planning and monitoring for persons with mental disorders with high utilization of psychiatric services. Results showed no differences between the two groups in days of hospitalization and readmission (primary outcome) and in compliance with aftercare, clinical outcome and quality of life (secondary outcome). The authors suggest that the low intensity of the intervention, which was comprised of only two sessions, could be a possible explanation for the lack of effect.

Likewise, in Switzerland no study results are currently available regarding systematic discharge planning. However, Warnke et al. [10] found that of 103 patients with a diagnosis of schizophrenia, about half were re-hospitalized within a year. Both clinical factors (such as not taking neuroleptic drugs), and social factors were associated with the risk of re-hospitalization. The authors concluded that prevention should consider both the illness and the social situation of the patient.

There are currently several projects throughout Switzerland that focus on improving outpatient care for high frequency service users with the aim to prevent unnecessary re-hospitalization. There is, however, less research being conducted in the area of discharge coordination and continuity of care from inpatient to outpatient settings for patients with lower utilization of inpatient services. A pilot project in Lausanne examined *case management de transition*, that is, case management to support the transition from inpatient to outpatient care [11]. The intervention in the study was specifically for patients who did not have a pre-existing outpatient care structure. Before this intervention, between 20% of patients from the schizophrenia unit and 70% of patients from the general psychiatric unit did not receive any psychiatric follow-up care after discharge from inpatient care. The results of the randomized controlled trial (RCT) have not yet been published.

This study, “Elements of integrated care: Post-hospitalization network coordination,” aims to meet the need for more studies examining the effect of discharge planning on low-frequency service users. Ipw is a suitable psychiatric institution for implementing a project on network coordination after discharge. At ipw, outpatient care services are available for high-frequency service users and thus continuity of care is improved. For low-frequency (or lower) service users, however, there is currently no comparable care intervention designed to improve continuity of care from the inpatient to the outpatient setting.

The aim of this randomized controlled trial is to test whether post-hospitalization network coordination can reduce the number of inpatient clinic days as compared to a control group that does not receive any additional outpatient support. Secondary outcome variables studied are social support, clinical symptoms, medication adherence, quality of life, and life situation and independence. Furthermore, *Quality of life* seems to be an important outcome variable, because for persons with mental disorders, improvements are often made not in terms of symptoms, but instead in terms of quality of life. *Clinical symptoms* are nonetheless included as an outcome variable in order to assess the effectiveness of the intervention on symptoms. We also examine the intervention’s effects on self-stigma and recovery orientation. Finally, medication adherence is assessed. Warnke et al. [10] showed that medication adherence reduces the probability of re-hospitalization. For this reason, differences in this area between the treatment group and control group are of interest.

### Research question

The main objective of this study is to investigate if the Post-Discharge Network Coordination Program (PDNC-P) achieves a reduction in the number of inpatient days between discharge and one-year follow-up. Secondary research questions are whether the coordination of the network improves social support, independence in daily life, and quality of life, reduces clinical symptoms, and improves medication adherence between discharge and one year follow-up.

Thus, the main hypothesis is: The treatment group will have fewer inpatient care days than the control group. Additional hypotheses are that at follow-up:

a The treatment group will show a higher level of independence in daily functioning than the control group.

b The treatment group will have more social support than the control group.

c The treatment group will show a larger decrease in psychiatric symptoms than the control group.

d The treatment group will show a larger increase in quality of life than the control group.

e Gender will not have an effect on outcome.

This project, “Elements of integrated care: post-discharge network coordination,” has been developed to study these hypotheses. This project is a subproject of the Zurich program for sustainable development of mental health services (ZInEP). ZInEP is a program which aims, with six diverse sub-projects, to concentrate on the interface between research and care.

## Methods/design

The study is conducted as a randomized controlled trial. Subjects are randomly assigned to either the control group or to the treatment group. Computer generated block randomization is used to assure both groups have the same number of subjects. Stratified block randomization is used for the psychiatric diagnosis of ICD-10, F1.

### Participants

The population under study is adult patients with psychiatric illness living in the Winterthur–Zurcher Unterland psychiatry care catchment area. A total of 160 patients are recruited in the first week of their inpatient stays at one of five ipw acute care units (two units at Psychiatrie-Zentrum Hard Embrach and two units at Klinik Schlosstal Winterthur).

Inclusion criteria:

1 The patient has had no more than three inpatient stays (including the stay in question) within the past three years.

2 The patient has a GAF score of 60 or lower.

3 The patient has the ability to provide written informed consent.

4 The patient’s age is between 18 and 64 years.

Exclusion criteria:

1 The patient has insufficient German language skills, which makes it difficult for the patient to provide usable data through questionnaires and interviews (assessment).

2 The patient is already being supported by a case manager.

3 The patient lives in a form of supportive housing.

### Ethical approval

This study has been approved on 17 Feb 2012 by the Cantonal Ethics Committee Zurich (reference nr. KEK-ZH-Nr. 2011-0175).

### Description of the intervention: post-discharge network coordination program (PDNC-P)

ZHAW and ipw collaborated to develop PDNC-P. The program aims to ease the transition between inpatient and outpatient care by coordinating a social support network that reduces negative relationships, promotes positive relationships, and helps patients integrate back into society.

The program assigns each patient to a social worker. Each patient meets with his or her social worker prior to discharge. The patient and the social worker collaboratively agree upon a close network of social support, a crisis plan, and the terms of program termination. The social worker visits the patient within the first week of discharge to support and monitor the patient’s adjustment to outpatient care and daily life. The social worker and at least two individuals from the social support network meet within the second week of discharge to discuss the patient’s personal goals and how the social network can help the patient better cope and adjust to daily life. A network representative is chosen to mediate between the patient and the social support network. After the home visit during the first week, the social worker schedules subsequent visits. The program is tailored according to the patient’s personal needs and the frequency of the visits is based on the patient’s progress and needs. The program concludes once the terms of termination are reached or after a maximum of three months after discharge from inpatient care. The social support network will continue to aid the patient after program termination.

### Description of “treatment as usual”

The control group receives Treatment as Usual (TAU), which means that the patients receive the same level of social work support that they would have received if they were not part of the study. As is usual, the responsible inpatient care unit psychiatrist decides whether social work support is a useful complement to the patient’s inpatient care. The main emphasis is on social work issues such as clarification of debt problems. Network coordination is also a part of regular social work, but the social work assistance ends when the patient is discharged from inpatient psychiatric care.

### Procedure

Patients are randomly assigned to either the treatment group or to the control group. The patients from the treatment group participate in PDNC-P upon discharge at ipw. Patients from the control group receive treatment as usual with no additional social support.

Both groups of patients are assessed at discharge (t_0_), 3 months after discharge (t_1_), and 12 months after discharge (t_2_). As shown in Table 1 patients’ psychological symptoms, independence in daily life, social support, and quality of life are assessed using standardized self-report instruments (MANSA, OQ-45, F-SozU, RAS, and ISMI) and observer-rated instruments (HoNOS, SOFAS, GAF, CSSRI-EU, Morisky-Score, and CGI), which will be discussed in more detail in the following sections. Recruitment began in September 2011 and all assessments will end 12 months after patient discharge.

**Table 1:** Measurements, listed according to person conducting the assessment and measurement time points

**Measurement time points**	**TG only**	**TG and CG**	
	**Social worker-rated**	**Self-rating**	**Assessor-rated**
Start of inpatient care: baseline = t_0_	CGI	MANSA	HoNOS
		OQ-45	SOFAS
		F-SozU K-14	GAF
		ISMI	Morisky score
		RAS	CSSRI-EU
3 months after discharge/at maximum: duration of network coordination = t_1_	CGI	MANSA	HoNOS
		OQ-45	SOFAS
		F-SozU K-14	GAF
			Morisky score
12 months after discharge: follow-up assessment = t_2_		MANSA	HoNOS
		OQ-45	SOFAS
		F-SozU K-14	GAF
		ISMI	Morisky score
		RAS	CSSRI-EU

Based on data available in clinical records, the assessors (project staff) verify whether a patient meets the subject inclusion criteria. If so, the assessors explain the study to the patients and invite them to participate. After being fully informed about study procedures, patients provide written informed consent. Patients are then assigned to either the treatment group (TG) or to the control group (CG) by block randomization. Subsequently, the baseline assessment at study entry (t_0_) is conducted for all participating subjects (TG and CG). As a rule, this assessment should be conducted prior to the participant’s first conversation with the social worker. At the end of this conversation, participants are informed whether they have been assigned to TG or CG. If a patient has been assigned to the TG, his or her first conversation with the social worker follows the baseline assessment. For patients in the CG, a social worker is called in only if the responsible physician deems the support of a social worker to be a useful complement to inpatient care (TAU). The TG receives support from the social worker for a maximum of three months after hospital discharge. The focus is on establishing and coordinating the outpatient care network. The CG receives social work treatment as usual (TAU) but no extra/additional social work support after discharge from inpatient care.

Patients in the TG are assessed by the social worker using the Clinical Global Impressions (CGI) Scale at the beginning of the intervention (t_0_) and again 3 months after discharge from inpatient care (t_1_). Finally, the study assessors conduct additional assessments of all subjects participating in the study (TG and CG) at three months (t_1_) and 12 months (t_2_) after discharge from inpatient care. For each assessment after discharge from inpatient care, the study participants are paid CHF 50 for expenses.

### Primary outcome measures

#### Frequency and duration of psychiatric hospitalization days

The number of inpatient days is monitored using the ipw database and the Client Socio-Demographic and Service Receipt Inventory-European Version (CSSRI-EU, German version) [12]. This Instrument assesses socio-economic characteristics and psychiatric care costs in five domains: Socio-demographics (age, gender, marital status, years of schooling, educational level, vocational training); living situation (alone, with relatives, etc.; type of accommodation, change in accommodation during the observation period); employment and income (employment status, occupational category, days of work lost, type and amount of state benefits); service receipt (hospital inpatient days, partly inpatient, outpatient, complementary services, criminal justice service contacts); medication profile (name/type of drug, dosage level, frequency (number and size of packaged medications purchased at pharmacy), price).

### Secondary outcome measures

#### Independence in daily functioning

The CSSRI-EU also gives information about the patient’s independence in daily functioning. For this topic, the Global Assessment of Functioning (GAF) Scale and the Social and Occupational Assessment Scale (SOFAS) are also used. The Global Assessment of Functioning (GAF) Scale [13] is a global assessment of psychological, social, and occupational functioning on a hypothetical continuum of mental–illness (100 to 1 in 10-point intervals): 100 to 91 = Superior functioning in a wide range of activities; 10 to 1 = Persistent danger of severely hurting self or others (e.g. recurrent violence) OR persistent inability to maintain minimal personal hygiene OR serious suicidal act with clear expectation of death.

The Social and Occupational Functioning Assessment Scale (SOFAS) [14] is structured similarly to the GAF; but does not require assessment of psychological symptoms. It rates social and occupational functioning on a continuum from excellent functioning to grossly impaired functioning (100 to 1 in ten-point intervals). Any impairment in social and occupational functioning that is due to physical and mental limitations resulting directly from medical conditions are also considered.

### Social support

Social support is measured by the F-SozU K-14, Fragebogen zur sozialen Unterstützung – Kurzform (K-14) (Social Support Questionnaire, Short Form) [15]. This instrument uses 14 items to assess the perceived social support of three central types (emotional support - 8 items; instrumental support - 3 items; social integration - 4 items). Norms are based on a representative German sample.

### Change in psychiatric symptoms

Symptom severity is rated by HoNOS-D, CGI and OQ-45. The Health of the Nation Outcome Scales (HoNOS) [16] is rated by the assessor. Twelve items differentially assess the severity of mental disorders in a five-point severity scale (ranging from no problem to severe problem); a total score can be computed by summing the 12 item ratings. Four subscales are measured: behaviour, impairment, symptoms, and social functioning. The Clinical Global Impressions Scale (CGI) [17] is rated by the social worker. This is a simple to administer rating scale for assessing the treatment/intervention response of patients with all groups of disorders, and it evaluates the following aspects: (1) illness severity; (2) improvement or worsening of the patient’s overall condition; (3) effectiveness of the treatment; (4) undesirable effects. An Efficacy Index of 0.25 (adverse effects outweigh therapeutic effects) to 4.00 (marked therapeutic effects, no adverse effects) relates therapeutic effects of the treatment to adverse effects of the treatment. The Outcome Questionnaire (OQ-45, German version) [18] is a self-rating instrument with 45 items in a five-point response scale (1 = never, 5 = always). It includes the dimensions of symptom distress (25 items), interpersonal relations (11 items), and social role (9 items). Furthermore, the OQ-45 contains items on alcohol abuse, suicide potential, and potential violence at work. In addition to three subscale scores, a total score reflecting overall level of mental disturbance can be used. The OQ-45 has been extensively researched and is widely used internationally (German norms are available).

### Quality of life

The Manchester Short Assessment of Quality of Life (MANSA) [19,20] is used to assess the quality of life by self-rating and consists of 16 items. Four items are answered yes/no and 12 items are answered on a seven-point response scale (very dissatisfied to very satisfied).

### Exploratory outcome measures

Medication adherence information is collected using the Morisky-Score. The Morisky Medication Adherence Scale (Morisky-Score) [21] is rated by assessor. It assesses 4 items regarding medication adherence using a yes/no response scale: (1) Do you ever forget to take your medications?; (2) Are you careless at times about taking your medications?; (3) Do you sometimes stop taking your medications when you feel better?; (4) Do you sometimes stop taking your drugs if they make you feel worse? Each “no” is scored 1 point and the score is the sum of the points for the four answers. The scale is: 4 = high adherence, 2 and 3 = medium adherence, 0 and 1 = low adherence.

The 29-item Internalized Stigma of Mental Illness Inventory [22] is used to measure self-stigma. Recovery orientation is assessed using the short 24-item version of the Recovery Assessment Scale [23,24].

### Power and sample size calculation

The main endpoint in this study is the reduction of inpatient stays (number of days in inpatient psychiatric care) during the study period. This will be calculated using the *t*-test for independent samples. According to Cohen [25], for an expected medium effect size, the number of participants in each group should be 64. Assuming carefully that approximately 25% of the patients will drop out of this study, 80 patients are recruited for each group in order to have a sufficient number of patients to test the efficacy of the intervention.

### Analysis

Outcome differences in primary and secondary outcome variables between the treatment group and control group will be analyzed statistically using the *t*-test. In particular, this applies to the main research question regarding differences in the number of days of inpatient care. As the distribution of the differences are likely to be skewed, we will check for normal distribution. If the distribution is not normal, the dependent variable will be treated as Poisson distributed or transformed to normal distributed using other transformations. If differences between TG and CG are revealed (for example concerning age), a covariance analyses will be proceeded.

To depict the changes of the individual measurements over time, the Generalized Linear, Mixed Effects Model will be used. When testing the hypotheses the level of significance to be used will be 5% (***α*** = 0.05). Alpha adjustment will be used. The statistical analysis of the data will be done using the computer program Statistical Package for the Social Sciences (SPSS).

## Discussion

Since the first weeks following inpatient treatment are very stressful for psychiatric patients and associated with a higher suicide risk [2], systematically planning discharge is very important. The strengths of the proposed research are threefold: First, we address a gap in the research by concentrating on patients, who are utilizing the clinic for only the first, second or third time. As opposed to high-frequency service users, this population has not been well-studied. Second, in our study of post-discharge network coordination, we focus on integration of the patients in their quotidian environment. Third, since social factors are associated with the risk of re-hospitalization [10], social workers try to promote the integration of the patient into their social networks, in addition to adjusting outpatient care and addressing daily life.

With duration of three months after discharge and several sessions with the participants, the post-discharge network coordination is relatively time intensive. A German study, [9] in which the intervention of discharge planning was reduced to two sessions did not show an effect of the intervention on number of admissions, length of stay, psychopathology, depression, and quality of life. It is possible that with psychiatric patients, a more extensive intervention is needed.

Our hope is that with the post-discharge network coordination, an effect on primary and secondary outcomes is achievable. Primarily, we expect that during the study time the hospitalization days of the intervention group will decline compared to the control group. As the research shows, effects of discharge planning on “soft” outcomes such as psychopathology and quality of life are harder to improve for people with mental illness (e.g. [26]). Secondarily, although network coordination is not a treatment of mental illness, an improvement in medication adherence and in adherence to the psychiatric outpatient treatment could have an indirect effect on psychopathology. Finally, we expect higher patient satisfaction with the perceived social support, which should lead to a better quality of life and improve psychopathology as well.

One component of the post-discharge network coordination is a social worker visiting with the patient at home within the first week of discharge. A limitation of the study, however, is that patients who do not agree to being seen at home cannot take part in the study.

There are two main limitations of the trial. First is the difficulty of recruiting psychiatric patients for a RCT-study [27]. For some patients, it is hard to understand what the study entails. Furthermore, it must be determined if a patient has the capacity for informed consent. To address this issue, first, we are always in contact with the patient’s therapist. Second, the information process and the written consent take place on two different days to give the patient time to consider and also to allow for the possibility that they may be in a better mental state on one of the two days. Second, we must anticipate a high drop-out rate. Sometimes patients who are feeling better do not agree to continue in the research process [Von Wyl A, Meier P, Chew Howard E, Andreae A: *Psychiatrisches Case Management im psychiatrischen Versorgungsumfeld: Qualitative Evaluation einer RCT-Studie.* In preparation]. Sometimes, the constitution of the patient makes a further assessment impossible, and they do not reply to various kinds of requests. Many seriously mentally ill patients cannot be located, due to frequent change of residence, not having a landline phone, or being homeless. All these aspects imply a challenge for RCT-studies with psychiatric patients. Nevertheless, this kind of research helps to identify the needs of these patients [28].

## Abbreviations

Ipw: Integrierte Psychiatrie Winterthur - Zürcher Unterland [Integrated Psychiatry Winterthur – Zuercher Unterland]; PDNC-P: Post-discharge network coordination program; ZInEP: Zürcher impulsprogramm zur nachhaltigen entwicklung der Psychiatrie [The Zurich program for sustainable development of mental health services].

## Competing interests

The authors declare that they have no competing interests.

## Authors’ contributions

AvW and AA are co-principal investigators. AvW and GH are the coordinating investigators and responsible for trial management. The study was initiated by AA, AvW, and GH, who designed the proposal and protocol in collaboration with WR and NR. WR is the director of the ZInEP program. AvW wrote and prepared the study protocol manuscript. All authors have read and contributed significantly to the manuscript. All authors have approved the final version of the manuscript.

## Pre-publication history

The pre-publication history for this paper can be accessed here:

http://www.biomedcentral.com/1471-244X/13/220/prepub

## References

[B1] Salize H Rössler W Becker T Mental health care in Germany: current state and trends Eur Arch Psychiatry Clin Neurosci 2007 257 92 103 10.1007/s00406-006-0696-9 17149540

[B2] Qin P Nordentoft M Suicide risk in relation to psychiatric hospitalization: evidence based on longitudinal registers Arch Gen Psychiatry 2005 62 427 432 10.1001/archpsyc.62.4.427 15809410

[B3] Bonsack C Schaffter M Singy P Charbon Y Eggimann A Guex P Etude qualitative des attentes d’un réseau sanitaire et social pour le suivi des troubles psychiatriques sévères dans la communauté Encéphale 2007 33 751 761 18357845 10.1016/j.encep.2006.06.001

[B4] Olfson M Mechanic D Boyer CA Hansell S Linking inpatients with schizophrenia to outpatient care Psychiatr Serv 1998 49 911 917 9661225 10.1176/ps.49.7.911

[B5] Bonsack C Pfister T Conus P Insertion dans les soins après une première hospitalisation dans un secteur pour psychose Encéphale 2006 32 679 685 17099591 10.1016/s0013-7006(06)76219-4

[B6] Nelson EA Maruish ME Axler JL Effects of discharge planning and compliance with outpatient appointments on readmission rates Psychiatr Serv 2000 51 885 889 10.1176/appi.ps.51.7.885 10875952

[B7] Meisler N Santos AB Rowland MD Smith S Molloy M Tyson S Bridging the gap between inpatient and outpatient providers using organizational elements of assertive community treatment Adm Policy Ment Health 1997 25 141 152 10.1023/A:1022234904523 9727213

[B8] Steffen S Kösters M Becker T Puschner B Discharge planning in mental health care: a systematic review of the recent literature Acta Psychiatr Scand 2009 120 1 9 10.1111/j.1600-0447.2009.01373.x 19486329

[B9] Puschner B Steffen S Völker KA Spitzer C Gaebel W Janssen B Klein HE Spiessl H Steinert T Grempler J Muche R Becker T Needsoriented discharge planning for high utilisers of psychiatric services: multicenter randomised controlled trial Epidemiology and Psychiatric Sciences 2011 20 181 192 10.1017/S2045796011000278 21714365

[B10] Warnke I Nordt C Ajdacic-Gross V Haug A Salize HJ Rössler W Klinische und soziale Risikofaktoren für Wiederaufnahmen in die stationäre Psychiatrie bei Patienten mit Schizophrenie: Eine Langzeitanalyse Neuropsychiatr 2010 24 243 251 21176705

[B11] Bonsack C Gibellini G Ferrari P Dorogi Y Morgan C Morandi S Koch N Le case management de transition: Une intervention à court terme dans la communauté après une hospitalisation psychiatrique Schweiz Arch Neurol Psychiatr 2009 160 246 252

[B12] Roick C Kilian R Matschinger H Bernert S Mory C Angermeyer MC Die deutsche Version des Client Sociodemographic Service Receipt Inventory (CSSRI-EU) Psychiatr Prax 2001 28 84 90 11605129 10.1055/s-2001-17790

[B13] Saß H Wittchen H-U Zaudig M Diagnostisches und Statistisches Manual Psychischer Störungen DSM-IV 1998 4 Göttingen: Hogrefe

[B14] Goldman HH Skodol AE Lave TR Revising Axis V for DSM-IV: a review of measures of social functioning Am J Psychiatry 1992 149 1148 1156 1386964 10.1176/ajp.149.9.1148

[B15] Fydrich T Sommer G Brähler E Fragebogen zur sozialen Unterstützung (F-SOZU) 2007 Göttingen: Hogrefe

[B16] Wing JK Beevor AS Curtis RH Park SB Hadden S Burns A Health of the Nation outcome scales (HoNOS),BJP Res Dev 1998 172 11 18 10.1192/bjp.172.1.11 9534825

[B17] Guy W Clinical global impressions ECDEU Assessment for psychopharmacology (Revised) 1976 Rockville, MD: National Institute of Mental Health 217 222

[B18] Lambert MJ Hannöver W Nisslmüller K Richard M Kordy H Fragebogen zum Ergebnis von Psychotherapie: Zur Reliabilität und Validität der deutschen Übersetzung des Outcome Questionnaire 45.2 (OQ-45.2) Z Klin Psychol Psychother 2002 31 1 40 47 10.1026//1616-3443.31.1.40

[B19] Priebe S Huxley P Knight S Evans S Applications and results of the Manchester short assessment of quality of life (MANSA) Int J Soc Psychiatry 1999 45 1 7 12 10.1177/002076409904500102 10443245

[B20] Kaiser W Isermann M Hoffmann K Huxley P Priebe S Zur Kurzerfassung subjektiver Lebensqualität Fortschritte der Neurologie und Psychiatrie 1999 67 413 425 10.1055/s-2007-994991 10548998

[B21] Morisky DE Green LW Levine DM Concurrent and predictive validity of a self-reported measure of medication adherence Med Care 1986 24 1 67 74 10.1097/00005650-198601000-00007 3945130

[B22] Ritsher JB Otilingam PG Grajales M Internalized stigma of mental illness: psychometric properties of a new measure Psychiatry Res 2003 121 31 49 10.1016/j.psychres.2003.08.008 14572622

[B23] Corrigan PW Giffort D Rashid F Leary M Okeke I Recovery as a psychological construct Community Ment Health J 1999 35 231 239 10.1023/A:1018741302682 10401893

[B24] Corrigan PW Salzer M Ralph RO Sangster Y Keck L Examining the factor structure of the recovery assessment scale Schizophr Bull 2004 30 1035 1041 10.1093/oxfordjournals.schbul.a007118 15957202

[B25] Cohen J Statistical power analysis for the behavioral sciences 1988 2 Hillsdale, NJ: Erlbaum

[B26] Dixon MD Goldberg R Iannone V Luchsted A Brown C Kreyenbuhl J Fang L Potts W Use of a critical time intervention to promote continuity of care after psychiatric inpatient hospitalization Psychiatr Serv 2009 60 4 451 458 10.1176/appi.ps.60.4.451 19339319

[B27] Kallert TW Schützwohl M Randomisierte kontrollierte Studien in der psychiatrischen Versorgungsforschung: Probleme der Durchführungspraxis Fortschr Neurol Psychiatr 2002 70 647 656 10.1055/s-2002-35856 12459946

[B28] Rössler W Löffler W Fätkenheuer B Riecher-Rössler A Does case management reduce the rehospitalization rate? Acta Psychatrica Scandinavica 1992 86 445 449 10.1111/j.1600-0447.1992.tb03295.x 1471537

